# Implementation and Analysis of Tightly Coupled Global Navigation Satellite System Precise Point Positioning/Inertial Navigation System (GNSS PPP/INS) with Insufficient Satellites for Land Vehicle Navigation

**DOI:** 10.3390/s18124305

**Published:** 2018-12-06

**Authors:** Yue Liu, Fei Liu, Yang Gao, Lin Zhao

**Affiliations:** 1 College of Automation, Harbin Engineering University, Harbin 150001, China; liuyuehrb@outlook.com (Y.L.); zhaolin@hrbeu.edu.cn (L.Z.); 2 Department of Geomatics, University of Calgary, Calgary, AB T2N 1N4, Canada; ygao@ucalgary.ca

**Keywords:** tightly coupled, PPP, MEMS-IMU, insufficient satellites

## Abstract

This paper implements and analyzes a tightly coupled single-frequency global navigation satellite system precise point positioning/inertial navigation system (GNSS PPP/INS) with insufficient satellites for land vehicle navigation using a low-cost GNSS receiver and a microelectromechanical system (MEMS)-based inertial measurement unit (IMU). For land vehicle navigation, it is inevitable to encounter the situation where insufficient satellites can be observed. Therefore, it is necessary to analyze the performance of tightly coupled integration in a GNSS-challenging environment. In addition, it is also of importance to investigate the least number of satellites adopted to improve the performance, compared with no satellites used. In this paper, tightly coupled integration using low-cost sensors with insufficient satellites was conducted, which provided a clear view of the improvement of the solution with insufficient satellites compared to no GNSS measurements at all. Specifically, in this paper single-frequency PPP was implemented to achieve the best performance, with one single-frequency receiver. The INS mechanization was conducted in a local-level frame (LLF). An extended Kalman filter was applied to fuse the two different types of measurements. To be more specific, in PPP processing, the atmosphere errors are corrected using a Saastamoinen model and the Center for Orbit Determination in Europe (CODE) global ionosphere map (GIM) product. The residuals of atmosphere errors are not estimated to accelerate the ambiguity convergence. For INS error mitigation, velocity constraints for land vehicle navigation are adopted to limit the quick drift of a MEMS-based IMU. Field tests with simulated partial and full GNSS outages were conducted to show the performance of tightly coupled GNSS PPP/INS with insufficient satellites: The results were classified as long-term (several minutes) and short-term (less than 1 min). The results showed that generally, with GNSS measurements applied, although the number of satellites was not enough, the solution still could be improved, especially with more than three satellites observed. With three GPS satellites used, the horizontal drift could be reduced to a few meters after several minutes. The 3D position error could be limited within 10 m in one minute when three GPS satellites were applied. In addition, a field test in an urban area where insufficient satellites were observed from time to time was also conducted to show the limited solution drift.

## 1. Introduction

The global navigation satellite system (GNSS)/inertial navigation system (INS) integrated system has been widely applied in land vehicle navigation due to the capability of the GNSS/INS integrated system to bridge the outage of GNSS. In recent years, the cost-effective GNSS/INS solution has drawn great attention from both academic and industrial areas with the development of a low-cost GNSS receiver and a low-cost microelectromechanical system (MEMS)-based inertial measurement unit (IMU). For GNSS, precise point positioning (PPP) can achieve submeter accuracy for land vehicle navigation using a single low-cost GNSS receiver. Low-cost MEMS-based IMUs have increasingly been applied in applications in recent years as well [1,2,3,4]. For GNSS application in land vehicle navigation, most satellite signals might be blocked when a land vehicle enters a GNSS-harsh environment (e.g., an urban canyon). In this case, the performance of the integrated system with insufficient satellites needs to be explored and analyzed.

Usually two ways are used to integrate GNSS and INS, namely a loosely coupled (LC) mode and a tightly coupled (TC) mode. LC integration directly fuses the outputs of GNSS and INS solutions, which is easier to implement [5,6,7]. In comparison, the TC mode utilizes one centralized filter to integrate two different types of raw measurements to optimize the solution, which is more complicated but can still be valid without enough satellites [1,8,9]. Therefore, the tightly coupled scheme was used in this paper to adopt insufficient GNSS measurements. Different types of nonlinear filters such as a particle filter [10,11,12] and an unscented Kalman filter [13] can be applied in GNSS/INS integration [14,15,16]. The most widely applied method is the extended Kalman filter. When a low-cost MEMS IMU is applied in the system, the main drawback is that the errors accumulate quickly in a short time. To overcome this, different constraints are usually applied to limit the quick drift of a MEMS-based IMU. In [5], the author developed a method by adding map information to improve performance. In [6,8], the authors utilized height constraints for land vehicle navigation. The most common and effective constraint for land vehicle navigation using MEMS-based IMUs is a velocity constraint, namely a nonholonomic constraint (NHC) [6,7,8,17,18]. 

Previous research has rarely provided details of TC performance using insufficient satellites with the application of NHCs. In [6], the author implemented and analyzed the TC mode using GPS satellites where the implementations were either standard positioning or differential GPS (DGPS). Nowadays, low-cost multi-constellation PPP using one receiver has become increasingly widely used, which has made land vehicle navigation in challenging environments more reliable and cost effective. Therefore, the analysis of the performance of PPP/INS using insufficient satellites is necessary and significant. This paper investigates and analyzes the performance of a tightly integrated system with different numbers of insufficient satellites using low-cost sensors. Field tests of the TC mode using a single low-cost receiver and a MEMS-based IMU with GPS/GLONASS satellites were conducted. With the results obtained, the improvement of the different number of satellites used is presented. Both long-term (several minutes) and short-term (less than one minute) results showed that although one satellite may have degraded the solution, more than two satellites applied improved the accuracy in most cases. When three GPS satellites were used, the drift could be largely reduced. Field test results in an urban area, where insufficient satellites were observed, also showed the limited drift of the integrated solution.

This paper is organized as follows. Section 2 introduces the fundamentals of GNSS, INS, and the integrated system. The INS error accumulation and NHC model are illustrated in Section 3. Section 4 presents the details of the tests conducted and the analysis of the results. The conclusions are in the last section.

## 2. Fundamentals of GNSS/INS Integration

In this section, the fundamentals of GNSS and INS are reviewed. Section 2.1 introduces the observation models of GNSS, whereas the theories for INS mechanization are illustrated in Section 2.2. Section 2.3 is about how two different systems are integrated in the Kalman filter.

### 2.1. GNSS PPP

GNSS has been widely applied in land vehicle navigation [19,20,21,22]. GNSS positioning accuracy can achieve centimeter to millimeter levels with appropriate methods [23,24]. Apart from differential positioning methods (e.g., real-time kinematic) in which the errors are removed to achieve high accuracy, precise point positioning becomes increasingly widely applied in land vehicle navigation using one single GNSS receiver [25]. In PPP, all the errors need to be modeled or reduced by applying precise products. For satellite orbit and clock errors, precise products are used to reduce the satellite-related errors. One thing that needs to be mentioned is that most satellite clock products are referred to ionosphere-free P1 and P2 code combination. Therefore, the differential C1-P1 and P1-P2code biases (DCB) have to be applied to correct the biases in the C1 code if a single-frequency receiver used. Advanced error calibration models are used to correct the relativistic effect, the Sagnac effect, and others [26](Liu, 2018). After the application of error models and precise products, the observation equation of each satellite for code and carrier-phase measurements could be formed as

(1) $$\begin{array}{l} P=\rho +cdt+d_{trop}+d_{iono}+\varepsilon \left(P\right)\\ \Phi =\rho +cdt+d_{trop}-d_{iono}+\lambda N+\varepsilon \left(\Phi \right) \end{array},$$

where *P* and Φ are the code and carrier-phase measurements, respectively, *ρ* is the geometric distance between satellite and receiver, *c* is the speed of light, *dt* is the receiver clock offset, *λ* is the carrier-phase wavelength, *N* is the carrier-phase ambiguity, *d_trop_* is the troposphere delay, *d_iono_* is the ionosphere delay, and *ε*(*) represents the corresponding noise of the measurement, including the multipath effects.

For the troposphere delay, the Saastamoinen model [27] is applied to correct the troposphere delay. Usually, the residual of the wet delay is also estimated after Saastamoinen correction to achieve high accuracy in dual-frequency PPP [28]. However, the troposphere delay is correlated with the height estimation, which leads to a longer convergence time [29]. To accelerate the convergence of PPP for land vehicle navigation, the residual of the troposphere delay was not estimated for this paper. For ionosphere delay, an ionosphere-free combination can be formed to remove the majority part if a dual-frequency GNSS receiver is used [30]. However, a single-frequency receiver was used for this paper. To correct the ionosphere delay, a global ionosphere map (GIM) product provided by the Center for Orbit Determination in Europe (CODE) was applied to reduce the ionosphere delay [31,32]. For this paper, only the position, receiver clock offset, and ambiguities were estimated. All the uncalibrated biases (e.g., fractional cycle biases and residuals of atmosphere delay) were partially absorbed into the ambiguities. This may have affected the final accuracy, but the convergence time was reduced.

### 2.2. INS Mechanization

The process of converting the inertial measurements into position, velocity, and attitude is called INS mechanization. In this paper, INS mechanization was conducted in the local-level frame (LLF). The general procedure of INS mechanization can be summarized as follows. First, based on the previous attitude and the angular rate measured by gyros, the current attitude can be calculated by compensating the angular rate caused by earth rotation and the movement of the LLF. Second, the measured specific force needs to be converted into the acceleration in the LLF based on the current attitude after compensation of the Coriolis force caused by the earth rotation and movement of the LLF. Third, the velocity can be computed by integration of the converted and compensated acceleration, whereas the position can be calculated by integration of the velocity. The procedure of mechanization could be given as [33]:

(2) $$\left[\begin{array}{c} {\dot{r}}^{l}\\ {\dot{v}}^{l}\\ {\dot{R}}_{b}^{l} \end{array}\right]=\left[\begin{array}{c} D^{-1}v^{l}\\ R_{b}^{l}f^{b}-\left(2\Omega_{ie}^{l}+\Omega_{el}^{l}\right)v^{l}+g^{l}\\ R_{b}^{l}\left(\Omega_{ib}^{b}-\Omega_{il}^{b}\right) \end{array}\right],$$

where the dot represents the time derivatives; the superscripts *l* and *b* represent the local-level frame and the IMU body frame; *r^l^* is the geodetic position; *v^l^* is the velocity in the LLF; $R_{b}^{l}$ is the rotation matrix from the body frame to the local-level frame; $\Omega_{ab}^{c}$ is the skew-symmetric matrix of the ration rate $\omega_{ab}^{c}$, which represents the rotation rate of frame *b* relative to frame *a* expressed in frame *c*; *g^l^* is the gravity in the LLF; $\Omega_{ib}^{b}$ is the gyro output of the IMU; and *f^b^* is the specific force measured by the accelerometers. *D*^−1^ is given as

(3) $$D^{-1}=\left[\begin{array}{ccc} 0 & \frac{1}{R_{M}+h} & 0\\ \frac{1}{(R_{N}+h)\cos\varphi } & 0 & 0\\ 0 & 0 & 1 \end{array}\right],$$

where *R_M_* is the meridian radius of the ellipsoid, *R_N_* is the normal radius of the ellipsoid, *h* is the altitude, and *φ* is the latitude.

Equation (2) indicates the procedures of mechanization in continuous space. However, in the actual implementation of the INS mechanization, the inertial measurements were discrete. Moreover, a quaternion was used to update the attitude instead of the rotation matrix, since a quaternion could avoid the singularity using Euler angles [34]. The details of conversion between the rotation matrix and the quaternion were illustrated in [33]. With discrete inertial measurements, the update of quaternion, velocity, and position could be rewritten as

(4) $$\begin{array}{l} q_{k+1}=q_{k}+\frac{1}{2}\left[2\left(\cos\frac{\theta }{2}-1\right)I+\frac{2}{\theta }\sin\frac{\theta }{2}S(\theta )\right]q_{k}\\ \Delta v^{l}=R_{b}^{l}f^{b}\Delta t-\left(2\Omega_{ie}^{l}+\Omega_{el}^{l}\right)V^{l}\Delta t+g^{l}\Delta t\\ v_{k+1}^{l}=v_{k}^{l}+\frac{1}{2}(\Delta v_{k}^{l}+\Delta v_{k+1}^{l})\\ \varphi_{k+1}=\varphi_{k}+\frac{1}{2}\frac{\left[v_{n,k}+v_{n,k+1}\right]}{R_{N}+h}\Delta t\\ \lambda_{k+1}=\lambda_{k}+\frac{1}{2}\frac{\left(v_{e,k}+v_{e,k+1}\right)}{\left(R_{N}+h\right)\cos\varphi }\Delta t\\ h_{k+1}=h_{k}+\frac{1}{2}\left(v_{u,k}+v_{u,k+1}\right)\Delta t \end{array},$$

where *q_k_* is the quaternion at the *k* epoch; ∆*v^l^* is the velocity increment in the LLF in one epoch; $v_{k}^{l}$ is the velocity in the LLF at the *k* epoch, which can be decomposed as *v_e,k_*, *v_n,k_*, and *v_u,k_* in the east, north, and up directions; *φ_k_*, *λ_k_*, and *h_k_* are the latitude, longitude, and altitude at the *k* epoch; ∆*t* is the time interval between two consecutive epochs; *θ* is the magnitude of the body rotation angle between two consecutive epochs; and *S*(*θ*) is the skew matrix, given as

(5) $$S(\theta )=\left[\begin{array}{cccc} 0 & \theta_{z} & -\theta_{y} & \theta_{x}\\ -\theta_{z} & 0 & \theta_{x} & \theta_{y}\\ \theta_{y} & -\theta_{x} & 0 & \theta_{z}\\ -\theta_{x} & -\theta_{y} & -\theta_{z} & 0 \end{array}\right],$$

where *θ_x_*, *θ_y_*, and *θ_z_* are the components of $\theta_{lb}^{b}$.

### 2.3. GNSS/INS Integrated Navigation

GNSS and INS measurements can be fused in a filter to provide continuous solutions when GNSS is in outage. In this paper, an extended Kalman filter (EKF) with an error state was applied. The process of EKF is shown as follows:

(6) $$\begin{array}{l} X_{k+1}^{-}=\Phi_{k+1,k}X_{k}+w_{k}\\ P_{k+1}^{-}=\Phi_{k+1,k}P_{k}\Phi_{k+1,k}^{T}+Q_{k}\\ K_{k+1}=P_{k+1}^{-}H_{k+1}^{T}{\left(H_{k+1}P_{k+1}^{-}H_{k+1}^{T}+R_{k+1}\right)}^{-1}\\ X_{k+1}=X_{k+1}^{-}+K_{k+1}(Z_{k+1}-H_{k+1}X_{k}^{-})\\ P_{k+1}=(I-K_{k+1}H_{k+1})P_{k+1}^{-} \end{array},$$

where $X_{k+1}^{-}$ is the prior state vector; *w_k_* is the process noise vector; $P_{k+1}^{-}$ is the predicted matrix of the estimation covariance of the state vector; Φ*_k+_*_1*,k*_ is the transition matrix; *Q_k_* is the covariance matrix of the process noise; *K_k+_*_1_ is the gain matrix; *H_k+_*_1_ is the design matrix; *R_k+_*_1_ is the covariance matrix of the measurements; *Z_k+_*_1_ is the measurement vector; *I* is the identity matrix; *X_k+_*_1_ is the posterior state vector; and *P_k_* is the corrected matrix of the estimation covariance of the state vector.

For INS, the errors of position, velocity, and attitude together with sensor biases are estimated in the state vector. For GNSS, the clock offsets and ambiguities also need to be included to be estimated in tightly coupled integration. The state vector for tight integration can be given as

(7) $$X={\left[\begin{array}{ccccc} \delta r & \delta v & \varepsilon & b_{f} & \begin{array}{ccc} b_{\omega } & cdt_{i} & N_{1∼n} \end{array} \end{array}\right]}^{T},$$

where δ* represents the errors; *r* and *v* represent the geodetic position and velocity, respectively; *ε* is the attitude error in the LLF; *b_f_* and *b_⍵_* are the accelerometer and gyro biases, respectively; *cdt_i_* represents the receiver clock offset for the *i*^th^ constellation; and *N*_1*~n*_ stands for the ambiguities for the *n* satellites. What needs to be mentioned here is that different receiver clock offsets need to be set when more than one constellation is used: Namely, *cdt_i_* stands for *m* unknowns when *m* constellations are used. Besides this, *N*_1*~n*_ stands for *n* unknowns where *n* is the number of satellites.

Through linearization of Equation (2) and modeling the inertial sensor biases as a first-order Gaussian–Markov process, the dynamic matrix of the state errors could be achieved as [33]:

(8) $$\dot{X}=F^{l}X+Gw,$$

where *F^l^* and *G* are the dynamic matrix and the shaping matrix, the details of which can be found in [33], and *w* is the driven noise. The transition matrix can be simplified as

(9) $$\Phi_{k+1,k}=e^{F^{l}\Delta t}\approx I+F^{l}\Delta t.$$

Since the errors are estimated in the state vector, an error-related measurement update is applied in the Kalman filter, namely the difference between the measured GNSS measurements by the GNSS receiver and the predicted GNSS measurements based on the INS results, which is shown as

(10) $$Z_{k+1}=\left[\begin{array}{c} \begin{array}{l} P_{GNSS}^{1}-P_{INS}^{1}\\ \Phi_{{}_{GNSS}}^{1}-\Phi_{{}_{INS}}^{1}\\ D_{GNSS}^{1}-D_{INS}^{1} \end{array}\\ \vdots \\ \begin{array}{l} P_{GNSS}^{n}-P_{INS}^{n}\\ \Phi_{{}_{GNSS}}^{n}-\Phi_{{}_{INS}}^{n}\\ D_{GNSS}^{n}-D_{INS}^{n} \end{array} \end{array}\right],$$

where *Z*_k+1_ is the measurement update in the Kalman filter; $P_{GNSS}^{n}$, $\Phi_{GNSS}^{n}$, and $D_{GNSS}^{n}$ represent the measured GNSS code, carrier-phase, and Doppler measurements of satellite *n*; and $P_{INS}^{n}$, $\Phi_{INS}^{n}$, and $D_{INS}^{n}$ are the predicted code, carrier-phase, and Doppler measurements of satellite *n* based on the INS mechanization results and the satellite state in this epoch. With *n* satellites observed, there are *3n* measurements available in the Kalman filter. The code and carrier-phase measurements can directly correct the current position errors, whereas the Doppler measurements correct the velocity. Since the velocity errors are coupled with the attitude errors and accelerometer errors, and the attitude errors are further coupled with gyro biases, all the errors in the state vector can be estimated in the Kalman filter. The design matrix for GNSS measurements is given as

(11) $$H=\left[\begin{array}{c} H_{P}\\ H_{\Phi }\\ H_{D} \end{array}\right]=\left[\begin{array}{cccccccc} SM & 0_{n\times 3} & 0_{n\times 3} & 0_{n\times 3} & 0_{n\times 3} & 1 & 0 & 0_{n\times n}\\ SM & 0_{n\times 3} & 0_{n\times 3} & 0_{n\times 3} & 0_{n\times 3} & 1 & 0 & I_{n\times n}\\ S^{\prime }M & SR_{l}^{e} & 0_{n\times 3} & 0_{n\times 3} & 0_{n\times 3} & 0 & 1 & 0_{n\times n} \end{array}\right],$$

where *H_P_*, *H*_Φ_, and *H_D_* are the code-related, carrier-phase-related, and Doppler-related components, respectively; and $R_{l}^{e}$ is the rotation matrix from the LLF to the earth-central earth-fixed (ECEF) frame. The details of *S*, *M*, and $S^{\prime }$ are given as follows:

(12) $$\begin{array}{c} S=\left[\begin{array}{ccc} {\frac{x^{r}-x^{s}}{\rho }}_{1} & {\frac{y^{r}-y^{s}}{\rho }}_{1} & {\frac{z^{r}-z^{s}}{\rho }}_{1}\\ \vdots & \vdots & \vdots \\ {\frac{x^{r}-x^{s}}{\rho }}_{n} & {\frac{y^{r}-y^{s}}{\rho }}_{n} & {\frac{z^{r}-z^{s}}{\rho }}_{n} \end{array}\right]\\ S^{\prime }={\left[\begin{array}{ccc} \left(\frac{1}{\rho }-\frac{dx^{2}}{\rho^{3}}\right)dv_{x}-\frac{dydx}{\rho^{3}}dv_{y}-\frac{dzdx}{\rho^{3}}dv_{z}{}_{1} & \cdots & \left(\frac{1}{\rho }-\frac{dx^{2}}{\rho^{3}}\right)dv_{x}-\frac{dydx}{\rho^{3}}dv_{y}-\frac{dzdx}{\rho^{3}}dv_{z}{}_{n}\\ -\frac{dxdy}{\rho^{3}}dv_{x}+\left(\frac{1}{\rho }-\frac{dy^{2}}{\rho^{3}}\right)dv_{y}-\frac{dzdy}{\rho^{3}}dv_{z}{}_{1} & \ddots & -\frac{dxdy}{\rho^{3}}dv_{x}+\left(\frac{1}{\rho }-\frac{dy^{2}}{\rho^{3}}\right)dv_{y}-\frac{dzdy}{\rho^{3}}dv_{z}{}_{n}\\ -\frac{dxdz}{\rho^{3}}dv_{x}-\frac{dydz}{\rho^{3}}dv_{y}+\left(\frac{1}{\rho }-\frac{dz^{2}}{\rho^{3}}\right)dv_{z}{}_{1} & \cdots & -\frac{dxdz}{\rho^{3}}dv_{x}-\frac{dydz}{\rho^{3}}dv_{y}+\left(\frac{1}{\rho }-\frac{dz^{2}}{\rho^{3}}\right)dv_{z}{}_{n} \end{array}\right]}^{T}\\ M=\left[\begin{array}{ccc} -(R_{n}+h)\sin\phi \cos\lambda & -(R_{n}+h)\cos\phi \sin\lambda & \cos\phi \cos\lambda \\ -(R_{n}+h)\sin\phi \sin\lambda & (R_{n}+h)\cos\phi \cos\lambda & \cos\phi \sin\lambda \\ \left[R_{n}(1-e^{2})+h\right]\cos\phi & 0 & \sin\phi \end{array}\right]. \end{array}$$

In Equation (12), *ρ* is the distance between the satellite and receiver; *dx*, *dy*, and *dz* are the coordinate differences; *dv_x_*, *dv_y_*_,_ and *dv_z_* are the velocity differences; (*x^s^*, *y^s^*, *z^s^*) represent the satellite coordinates; and (*x^r^*, *y^r^*, *z^r^*) are the receiver coordinates that were obtained from the INS mechanization. All the components are given as

(13) $$\begin{array}{l} \rho =\sqrt{{\left(x^{r}-x^{s}\right)}^{2}+{\left(y^{r}-y^{s}\right)}^{2}+{\left(z^{r}-z^{s}\right)}^{2}}\\ dx=x^{r}-x^{s}\\ dy=y^{r}-y^{s}\\ dz=z^{r}-z^{s}\\ dv_{x}=v_{xr}-v_{xs}\\ dv_{y}=v_{yr}-v_{ys}\\ dv_{z}=v_{zr}-v_{zs} \end{array}.$$

## 3. INS Error Accumulation and Mitigation

Uncompensated inertial sensor errors contribute to the quick accumulation of position error. According to [33,35], accelerometer biases introduce an error proportional to time *t* in the velocity that leads to position errors proportional to the square of time, given as

(14) $$\delta v=\int b_{f}dt=b_{f}t\delta p=\int vdt=\int b_{f}tdt=\frac{1}{2}b_{f}t^{2}.$$

The gyro biases introduce an angle error (in pitch or roll) proportional to time *t*, which results in acceleration incorrectly projected on the horizontal channels. Similarly, this acceleration error caused by misalignment finally introduces errors in velocity and position, given as

(15) $$\begin{array}{l} \delta \theta =\int b_{\omega }dt=b_{\omega }t\delta a=g\sin(\delta \theta )\approx g\delta \theta \approx gb_{\omega }t\\ \delta v=\int gb_{\omega }tdt=\frac{1}{2}gb_{\omega }t^{2}\delta p=\int \delta vdt=\frac{1}{6}gb_{\omega }t^{3} \end{array}.$$

Equations (14) and (15) indicate that the mechanization errors increase quickly without the correction of inertial sensor errors. With GNSS measurements available, the inertial sensor errors together with the velocity and position errors can be estimated. However, when the land vehicle enters harsh environments where not enough satellite signals can be received, a MEMS-IMU solution drifts fast in a short time due to the dramatically accumulated inertial sensor errors. Therefore, it is necessary to apply other constraints to limit the quick drift of INS solutions. For land vehicle navigation, the velocity in the right and up directions of the land vehicle is close to zero most of the time, which can be used as a nonholonomic constraint (NHC) for MEMS IMUs if the land vehicle is aligned with the IMU. These constraints can directly correct part of the velocity and attitude components. Since the inertial sensor errors are coupled with velocity and attitude errors, they can be estimated as well. All these corrections contribute to reducing drift. The relationship between the velocity in the body frame and the LLF can be given as

(16) $$\begin{array}{cc} v^{b} & =R_{l}^{b}v^{l}\\ \delta v^{b} & =-R_{l}^{b}\Psi v^{l}+R_{l}^{b}\delta v^{l}\\ & =R_{l}^{b}V^{l}\varepsilon +R_{l}^{b}\delta v^{l}\\ Z_{NHC} & =0_{3\times 1}-v^{b} \end{array},$$

where *v^b^* is the velocity in the body frame; *V*^l^ is the skew-symmetric matrix of the velocity *v*^l^ in the LLF; and Ψ is the skew-symmetric matrix of the attitude error *ε*. *Z_NHC_* is the NHC measurement, which is the difference between the actual and calculated velocity in the IMU body frame. The right and up velocity is close to zero, whereas the actual forward velocity is unknown. In this equation, the forward velocity is set as zero as well, which is not the actual situation in land vehicle navigation. Therefore, the uncertainty of the forward velocity error measurement is set as a large value, which makes it contribute little in the Kalman filter. According to Equation (16), the velocity in the body frame is observable for a attitude and velocity errors in the state vector. Therefore, with the NHC, the quick drift can be reduced to some extent. Equations (10) and (16) can be combined to provide the measurement update in a harsh environment. The measurement update and the corresponding design matrix can be expanded as

(17) $$Z_{k+1}=\left[\begin{array}{c} P_{GNSS}^{1}-P_{INS}^{1}\\ \vdots \\ P_{GNSS}^{n}-P_{INS}^{n}\\ \Phi_{{}_{GNSS}}^{1}-\Phi_{{}_{INS}}^{1}\\ \vdots \\ \Phi_{{}_{GNSS}}^{n}-\Phi_{{}_{INS}}^{n}\\ D_{GNSS}^{1}-D_{INS}^{1}\\ \vdots \\ D_{GNSS}^{n}-D_{INS}^{n}\\ -v^{b} \end{array}\right]\;H=\left[\begin{array}{c} H_{P}\\ H_{\Phi }\\ H_{D}\\ H_{NHC} \end{array}\right]=\left[\begin{array}{cccccccc} SM & 0_{n\times 3} & 0_{n\times 3} & 0_{n\times 3} & 0_{n\times 3} & 1 & 0 & 0_{n\times n}\\ SM & 0_{n\times 3} & 0_{n\times 3} & 0_{n\times 3} & 0_{n\times 3} & 1 & 0 & I_{n\times n}\\ S^{\prime }M & SR_{l}^{e} & 0_{n\times 3} & 0_{n\times 3} & 0_{n\times 3} & 0 & 1 & 0_{n\times n}\\ 0_{3\times 3} & R_{l}^{b} & R_{l}^{b}V^{l} & 0_{3\times 3} & 0_{3\times 3} & 0_{3\times 3} & 0_{3\times 3} & 0_{3\times 3} \end{array}\right].$$

## 4. Field Tests and Results

To investigate the performance of tightly coupled GNSS/INS with insufficient satellites, field tests with low-cost MEMS IMUs and single-frequency receivers were conducted in Calgary. In these tests, a Ublox NEO M8U evaluation kit (EVK-M8U) (Thalwil, Switzerland) was used to provide the raw GNSS and IMU datasets. The EKV-M8U can provide 100 Hz raw inertial measurements with an IMU internal clock, which needs to be synchronized with the GNSS measurements. Given that the EVK-M8U also provides up to 20 Hz of a high-rate solution with the GPS time, the INS measurement can be tagged with GPS time by interpolating the high-rate solution time.

**Figure 1:**
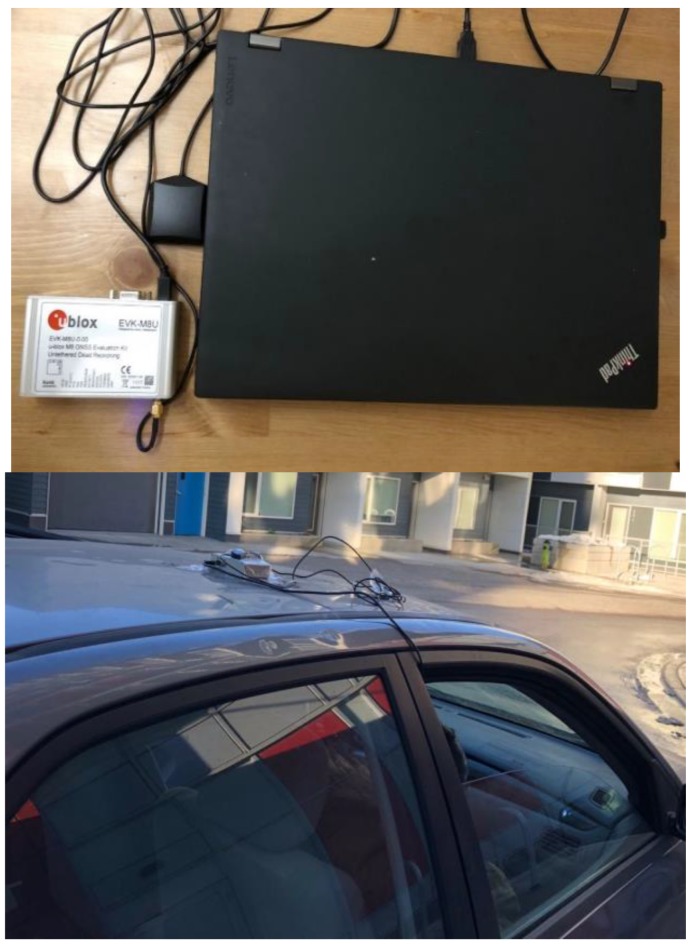
Field test equipment and setup: One Ublox EVK-M8U and one patch antenna mounted on the vehicle roof were used in the tests. A laptop was used to store the datasets. The antenna was mounted directly on the EVK-M8U, and the level arm was neglected.

The EVK-M8U was mounted on the roof of a land vehicle, as shown in Figure 1. The Ublox patch antenna was used to receive GNSS signals, and all the datasets were stored in a laptop. Single-frequency PPP algorithms were adopted to process the GNSS measurements. The troposphere error was corrected using the Saastamoinen model [27], and the residuals after correction were not estimated in the state vector to accelerate the convergence of single-frequency PPP. Similarly, the ionosphere errors were only corrected using CODE GIM products [31] without estimation in the state vector. The elevation mask was set as 10 degrees to exclude GNSS measurements with large noise and multipaths. When both GPS and GLONASS measurements were used, the weight of the GPS measurements was 1.5 times that of the GLONASS measurements. In the processing of inertial datasets in this paper, NHC was applied to limit the quick drift of low-cost MEMS IMU. A first-order Gaussian–Markov process was used to model the inertial sensor errors, with a correlation time of 15 min and a standard deviation of 10 mg and 40°/h for accelerometers and gyros, respectively.

**Figure 2:**
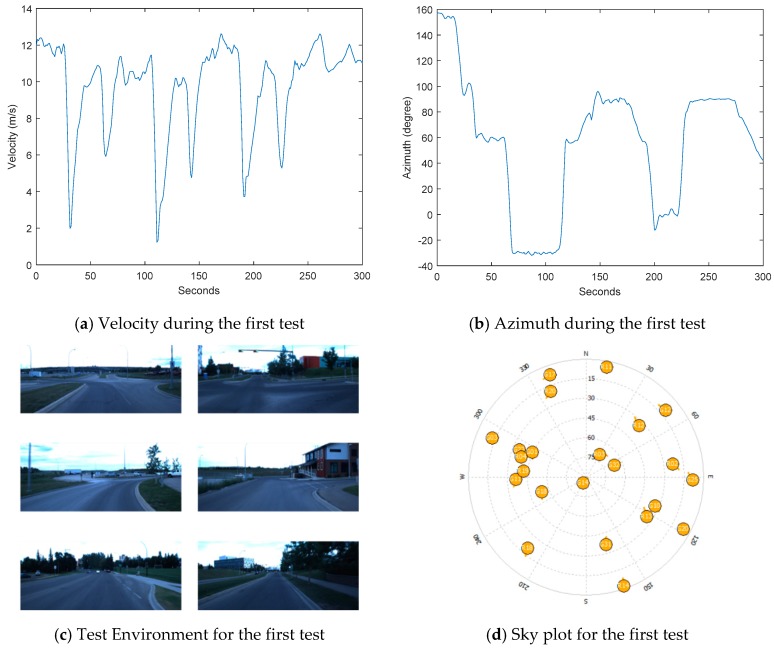
Velocity, azimuth, environment, and sky plot of the satellites for the first test.

The first field test was around the Alberta Children’s Hospital, starting from a parking lot nearby. Most of the time, the environment was open sky except for buildings along the road from time to time. After moving for about 4 min in open sky to make ambiguities and inertial sensor biases converge, the GNSS measurements were excluded manually. The total time for simulated GNSS outage was 5 min, with a total horizontal distance of about 2.9 km. The land vehicle velocity, moving azimuth, test environment, and the sky plot of satellites are shown in the subplots of Figure 2. When the land vehicle was doing maneuvers or meeting pedestrians, it slowed down, which could be seen from the velocity dropping from time to time. Zero velocity update (ZUPT) was not applied during the test.

**Figure 3:**
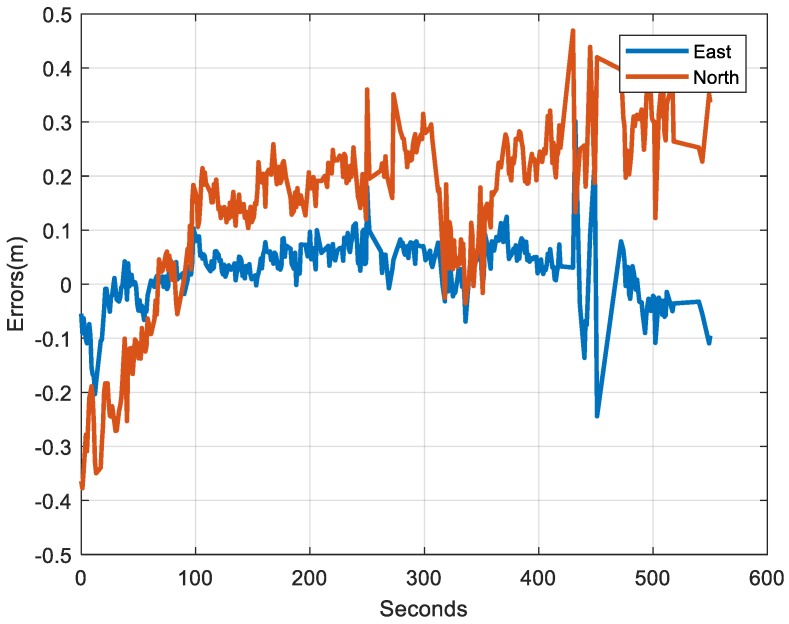
Horizontal errors of single-frequency precise point positioning (PPP).

There were 5 maneuvers during the test, and the satellites could be observed at most times except for one epoch without GNSS measurements. The reference used was the single-frequency PPP with GPS and GLONASS. The horizontal accuracy of the PPP solution was at the submeter level, which can be seen in Figure 3. Shown in Figure 3 is the east and north error of the PPP, compared to the real-time kinematic (RTK) fixed solution with a base station set up at the roof of the University of Calgary Engineering Building with total open sky. The reason the RTK fixed solution was not directly used as the reference is that the RTK solution could not be fixed at some epochs, whereas the PPP solution was available at most times in this dataset. The PPP solution was accurate enough to evaluate the solutions with insufficient satellites since the trajectories without enough satellites drifted over time.

**Figure 4:**
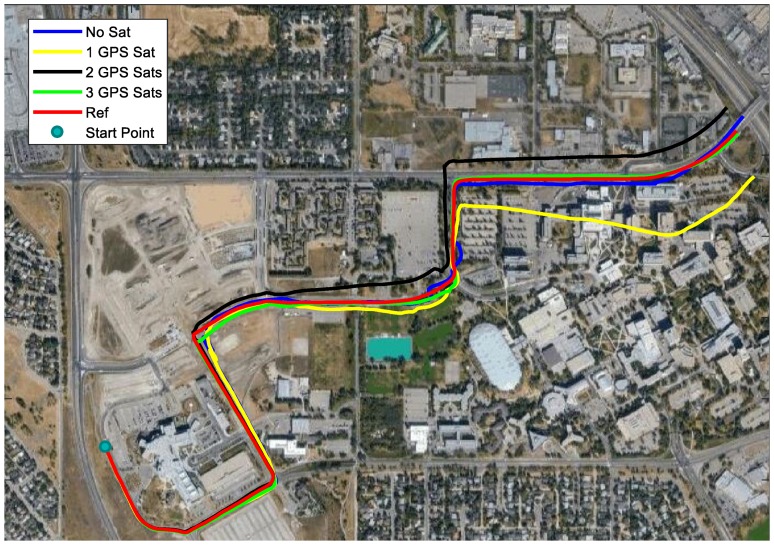
Trajectories using different numbers of GPS satellites.

Since the satellites with a lower elevation were more likely to be blocked in applications, only the satellites with a higher elevation were applied in the partial GNSS outage simulation. Specifically, the usage of the satellites in the GNSS outage simulation was as follows. According to the sky plot in Figure 2, G14 was selected for 1 satellite used; G14 and G32 were selected for 2 satellites used; and G14, G32, and G18 were selected for 3 satellites used. The moving trajectories with different numbers of satellites used are plotted in different colors in Figure 4. The trajectories without enough satellites were close to the reference in the first several seconds and started to become worse after the second maneuver.

**Figure 5:**
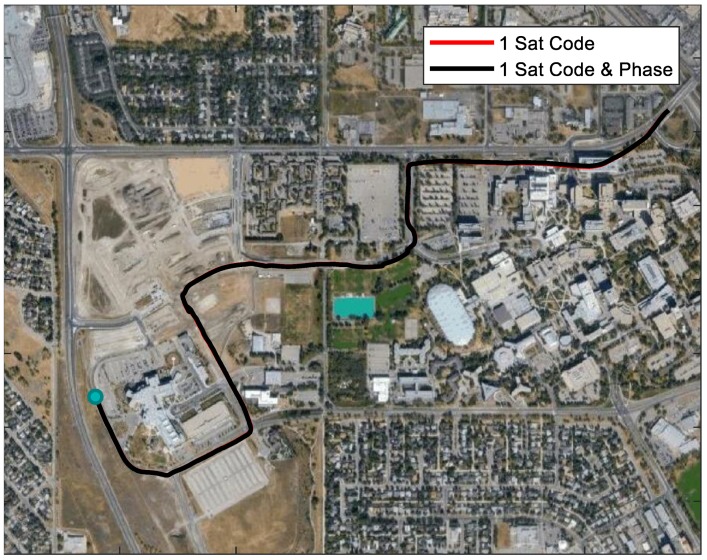
Trajectories of using G32.

The trajectories in Figure 4 were obtained using GPS code and carrier-phase measurements, as illustrated in the previous section. When enough satellites were used, the application of carrier-phase measurements resulted in a better solution because of much smaller noise. However, in the scenario where not enough satellites could be observed, the processing results of applying code only were similar to utilizing both code and carrier-phase measurements. This was due to the drift being much larger than improvements of phase measurements with small noise. The same geometry of code and phase measurements made the extra phase measurements contribute little to the solution. Besides, ambiguities had to be estimated with phase measurements used, resulting in equal measurement redundance. Figure 5 shows the very similar trajectories obtained by using 1 GPS satellite code only and both code and carrier-phase measurements. For the rest of this paper, both code and carrier-phase measurements were used in processing.

When more GNSS measurements were used, better performance could be expected. With three GPS satellites applied, the drift of the trajectory was quite small, which only occurred near the third maneuver. One thing that needs to be mentioned is that the worst trajectory in Figure 4 was the one with one satellite used. With one satellite used, three extra unknowns (receiver clock offset, drift, and phase ambiguity) were introduced, which equaled the GNSS measurements introduced. It is possible that the result with one satellite was worse than with no satellites used at all. If G32 was selected instead of G14 for one satellite used, the trajectory was better than G14 used, which is shown in Figure 5. Therefore, improvements could not be expected in all cases. Based on this 5-min test, only 3 satellites used could outperform the solution with no satellites used. This might have been caused by the inaccurate estimation of the inertial sensor biases that worsened the solution.

**Figure 6:**
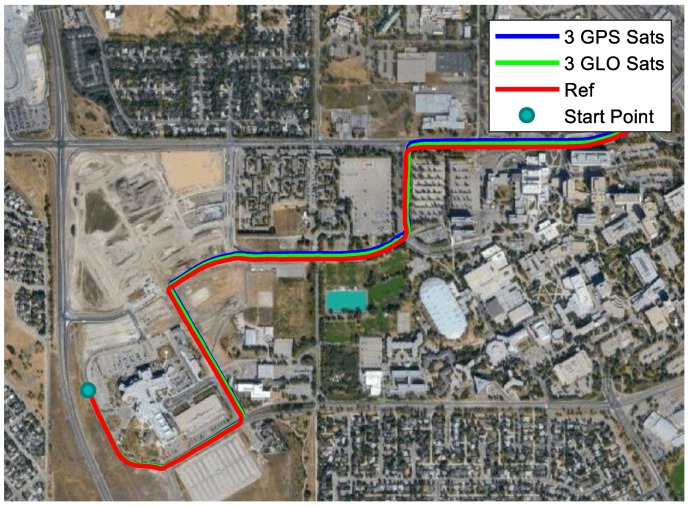
Trajectories of using GPS and GLONASS with similar geometric distributions.

**Figure 7:**
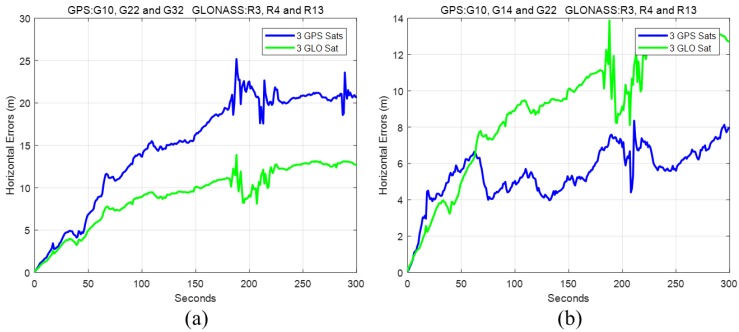
Horizontal errors with 3 GPS and 3 GLONASS satellites. (**a**) GPS satellites: G10, G22 and G32; (**b**) GPS satellites: G10, G14 and G22.

Apart from the application of GPS measurements, GLONASS measurements were also processed to simulate the GNSS outage. Usually, more than ten GPS and GLONASS satellites could be observed, which made reliable navigation possible in a challenging environment (e.g., an urban canyon). Although GPS satellites may be blocked in challenging environments, it is likely that a few reliable satellites are available with multiple constellations. For the same test dataset, results using both GPS and GLONASS satellites were also presented and compared to GPS-only solutions. With GLONASS measurements used, the GLONASS receiver clock offset and clock drift were also introduced into the state vector. In other words, for Equation (7), 2 GPS-related and 2 GLONASS-related unknowns together with *N* ambiguities (*N* is the number of carrier-phase measurements) also need to be estimated. The total number of GPS and GLONASS satellites has to be more than five to avoid drift. In the following discussion of insufficient satellites used, the results of using different numbers of GPS and GLONASS satellites are presented. In practical applications, the satellites with higher elevations are less likely to be blocked. Before the discussion of satellite combinations with high elevations, the performances of GPS and GLONASS of similar geometric distributions are investigated. Based on the sky plot in Figure 2, G10, G22, and G32 had a similar distribution to R3, R4, and R13. The trajectories obtained by the three GPS satellites and three GLONASS satellites are provided in Figure 6, and the horizontal errors are shown in Figure 7a.

It can be seen that the error growth trends for GPS and GLONASS were very similar to each other. Since the geometric distribution could not be exactly the same, in this case the performance of GLONASS was better than GPS. However, if we chose G14 instead of G32, the horizontal error was largely reduced, as shown in Figure 7b. Therefore, the geometric distribution has a great impact on the performance when insufficient satellites can be observed.

**Figure 8:**
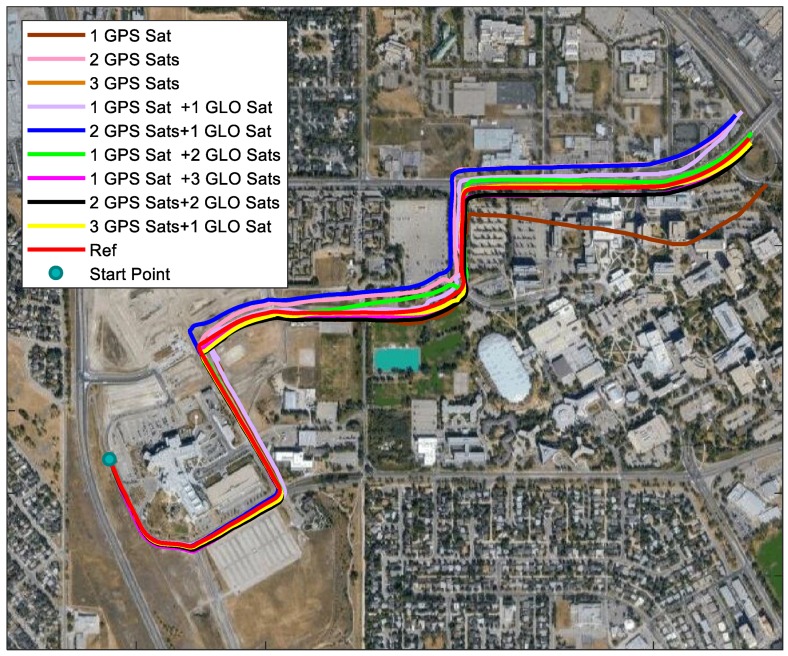
Trajectories of using different GPS and GLONASS satellites.

As mentioned before, satellites with higher elevations are less likely to be blocked. Therefore, the performance of insufficient satellites with high elevation needs to be discussed. Different combinations of satellites used are as follows: (1) 1 GPS satellite and 1 GLONASS satellite, (2) 1 GPS satellite and 2 GLONASS satellites, (3) 2 GPS satellites and 1 GLONASS satellite, (4) 1 GPS satellite and 3 GLONASS satellites, (5) 2 GPS satellites and 2 GLONASS satellites, (6) 3 GPS satellites and 1 GLONASS satellites. Specifically, the satellites used are as follows: G14, G32, and G18 were selected for GPS satellites, and R3, R19, and R4 were selected for GLONASS satellites. Combined with the previous GPS-only results, the trajectories with different satellites used are plotted in Figure 8.

**Figure 9:**
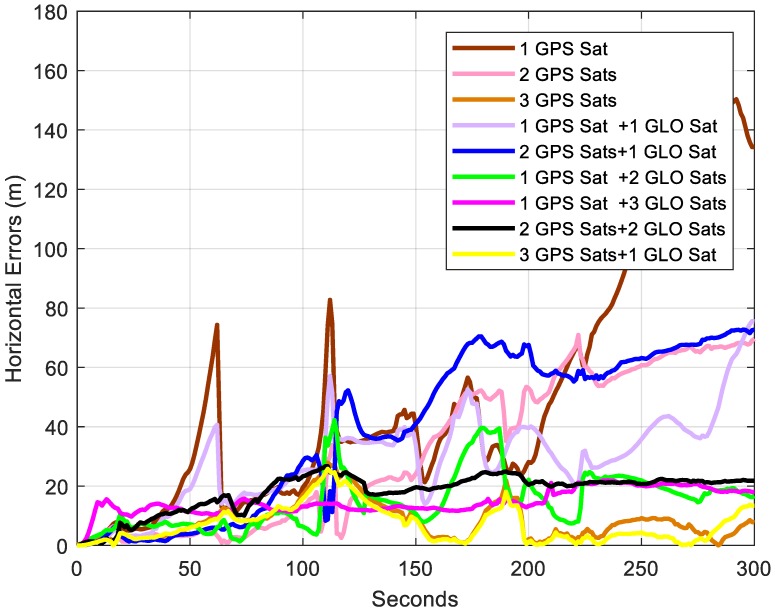
Horizontal errors of trajectories using different satellites.

In Figure 8, solutions with no less than 3 satellites were close to the reference all the time. To see the performance of solutions using different satellites more clearly, the horizontal errors of the trajectories are shown in Figure 9. All the solutions suffered drift over time due to insufficient satellites. Generally, with more satellites used, less drift could be expected. When more than 3 satellites were applied, the horizontal errors were less than 40 m regardless of the GPS and GLONASS combinations, except for 2 GPS satellites and 1 GLONASS satellite. Among all the solutions, the one with 3 GPS satellites and 1 GLONASS satellite outperformed others, with a horizontal error less than 20 m in most time. To have a better understanding of the solution performance, the maximum errors, root mean square (RMS), and relative horizontal position error are summarized in Table 1. It is clear that when only one GPS satellite was used, the performance was the worst, compared to other solutions. With 2 satellites applied, 2 GPS satellites were supposed to outperform the 1 GPS satellite and 1 GLONASS satellite since with two different constellations, one more unknown was introduced, compared to one constellation. However, the results showed that 1 GPS and 1 GLONASS performed better. Therefore, the distribution of satellites affected the performance when insufficient satellites were used. When 3 satellites were used, 3 GPS satellites generated the best solution. With 4 satellites applied, 3 GPS satellites and 1 GLONASS satellite outperformed other solutions.

In some circumstances (e.g., in underground parking or tunnels), no GNSS satellites can be observed for a while. However, when the land vehicle was in a GNSS-challenging environment such as an urban canyon, in the most cases, the time for insufficient satellites did not last for several minutes, especially when multi-constellation GNSS measurements were applied. It was more common that insufficient satellites were observed for several seconds from time to time with high building blockages. Therefore, it was necessary to analyze the performance of solutions using insufficient satellites in a short time. To do this, the simulated blockage started at 4, 5, 6, 7, and 8 min, and the accuracy of the solutions at the first 3 s, 10 s, 30 s, and 60 s, was evaluated. The 3D RMS of the solutions using different satellites is summarized in Table 2. It can be seen that, in the short term, more satellites generated better results except for 3 GLONASS satellites used.

**Figure 10:**
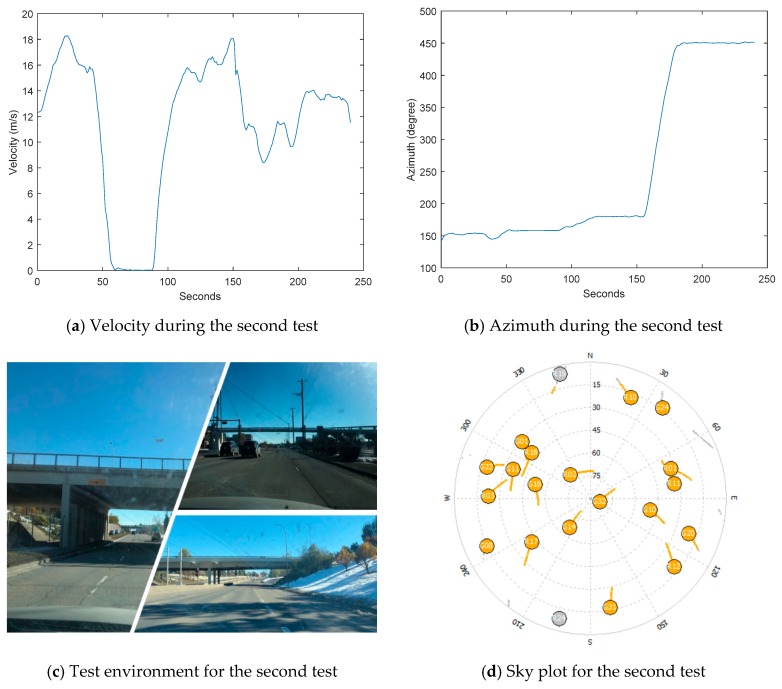
Velocity, azimuth, environment, and sky plot of the satellites for the second test.

To further analyze the tightly coupled results of GNSS and INS with insufficient satellites, another dataset was collected on the highway in Calgary. Similarly to the first test, to make the ambiguities and inertial sensor biases converge, the GNSS outage was simulated after 290 s of moving. Several maneuvers were made before the simulation of the GNSS outage in order to make the azimuth estimation converge. This time, the simulated GNSS outage time was 4 min in total, and the total distance was about 2.7 km. The land vehicle passed through 3 bridges during the test where satellites were all blocked for one or two seconds. The velocity, azimuth, test environment, and satellite sky plot are shown in Figure 10. The velocity of the land vehicle dropped to zero between 50 s and 100 s due to the traffic light. ZUPT was not applied during this period. The GPS satellites and GLONASS satellites used were G32, G14, and G18, and R2, R17, and R18. When some satellites were not available at some epochs, other satellites replaced them temporally based on the elevation.

**Figure 11:**
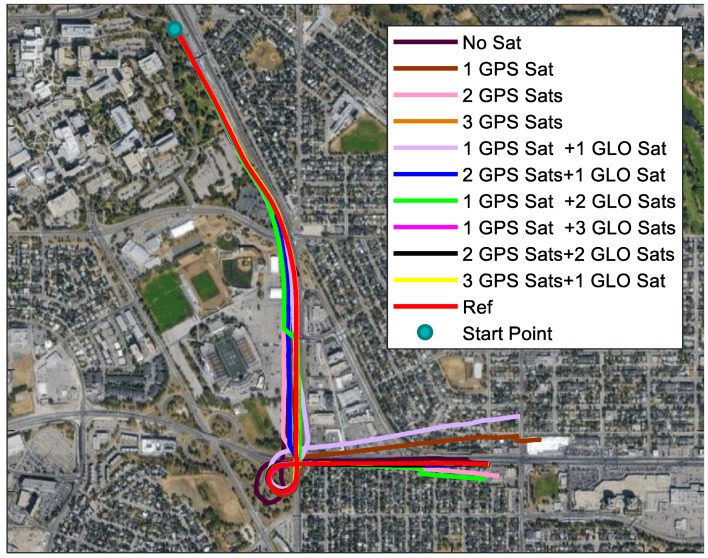
Trajectories of using different GPS and GLONASS satellites.

**Figure 12:**
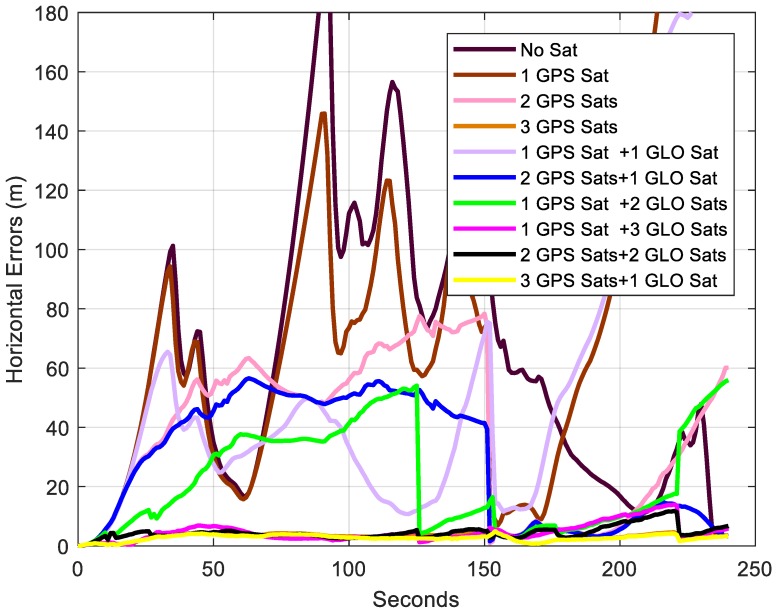
Horizontal errors of trajectories using different satellites.

The trajectories and the horizontal errors using different satellites are plotted in Figure 11 and Figure 12, respectively. The sudden accuracy change around 150 s was due to passing through one bridge, and moving direction change after that. In addition to this, the trajectory using 1 GPS satellite and 2 GLONASS satellites had another sudden change at about 125 s. This is because one of the GLONASS satellites used was changed because of the elevation change. The second-highest GLONASS satellite was changed from R17 to R18. Therefore, the performance was highly related to the orientation of the satellites used. The RMS, maximum errors, and relative horizontal positioning errors of each trajectory are summarized in Table 3. Generally, better results could be expected with more satellites used. With one satellite used, the horizontal accuracy was not improved. When 2 or more satellites, especially GPS satellites, were applied, the horizontal accuracy had an evident improvement. The largest drift was only 5 m when 3 GPS satellites were applied.

Similarly to the previous dataset, the GNSS outage was simulated at different times to calculate the 3D errors in 3 s, 10 s, 30 s, and 60 s. To avoid the land vehicle static period, the other two outages were started at 120 s and 180 s. The RMS of solutions using different satellites is summarized in Table 4. The 3D position accuracy of this dataset in short periods of outage was not as good as the previous one.

**Figure 13:**
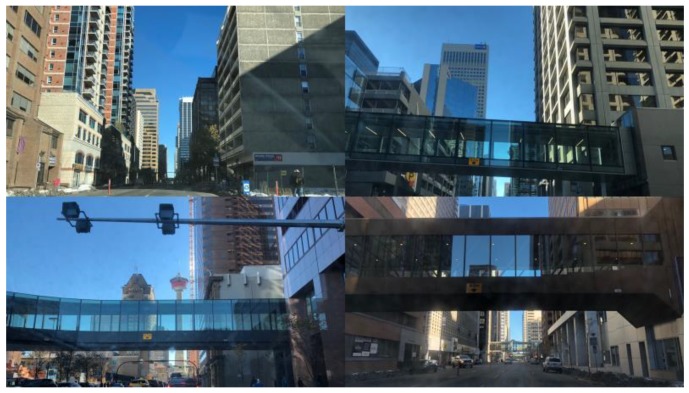
Test environment in an urban area: In this test, the environment was typical urban canyon with buildings along the road and sky bridges from time to time.

**Figure 14:**
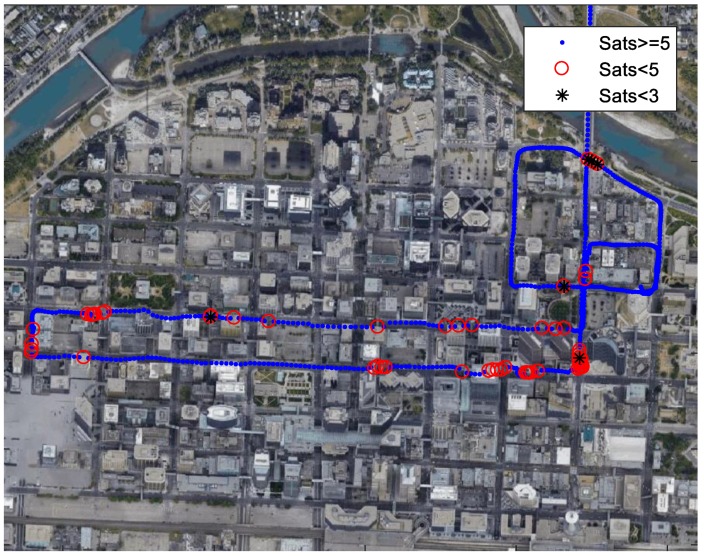
Test trajectory in Calgary’s downtown.

In addition to the simulated GNSS outage, a field test in Calgary’s downtown was also conducted. During this test, insufficient satellites were observed from time to time because of the building blockage. The test started in an open sky environment outside the downtown. The duration in the downtown was about 9 min, with high buildings along the road. The test environment was shown in Figure 13. Single-frequency GPS and GLONASS PPP and MEMS-based IMUs were integrated. The trajectory of this test is plotted in Figure 14. The red circles represent the points with insufficient satellites (less than five satellites). The epochs with less than 3 satellites are denoted by a black star. The total number of epochs with less than 5 satellites was 62, and the longest duration of insufficient satellites lasted for 7 s. The number of epochs with less than 3 satellites was 6. Although there was no reference for this test in the downtown, the drift without enough satellites was limited since the trajectory was still on the road.

## 5. Conclusions

This paper implemented the tightly coupled single-frequency GNSS PPP/INS with NHC using a low-cost GNSS receiver and a MEMS-based IMU. The field results showed that in long-term GNSS outages (several minutes), the horizontal drift without GNSS could be limited to 200 m with movement close to 3 km. Generally, with more satellites used, the accuracy of solutions can be improved. When one satellite was used, the accuracy might not be improved, compared to no satellites used at all, because of no increase in measurement redundance. The distribution of the satellites affects the solution accuracy. Since the NHC provides velocity constraints in the right and up directions in the body frame, the accuracy can be improved with better estimation of forward velocity. Higher velocity is likely to generate worse results, comparing the two datasets. For both datasets collected, the horizontal accuracy could be reduced to a few meters with three GPS satellites applied.

In short-term GNSS outages (less than one minute), the position errors were less than 1 m within 3 s. Generally, the 3D position accuracy was improved with more satellites used as well. The improvement was more evident with longer periods of GNSS outages. Within half a minute, most of the errors were less than 10 m with more than two satellites applied. With three GPS satellites used, the RMS of the position error was less than 7 m within 1 min. The trajectory without enough satellites in the urban area was still on the road, which indicates the drift was limited.

## Figures and Tables

**Table 1:** Summary of horizontal errors using different satellites. GLO: GLONASS; sat: satellite.

Satellites Used	RMS (m)	Maximum Error (m)	Relative Horizontal Position Error
No sat	29.153	77.435	1.00%
1 GPS sat	70.147	165.962	2.41%
2 GPS sats	40.938	70.918	1.40%
3 GPS sats	10.345	27.859	0.35%
1 GPS sat & 1 GLO sat	32.721	75.575	1.12%
2 GPS sats & 1 GLO sat	47.501	72.745	1.63%
1 GPS sat & 2 GLO sats	17.499	42.236	0.60%
1 GPS sat & 3 GLO sats	15.264	21.925	0.52%
2 GPS sats & 2 GLO sats	19.184	26.708	0.66%
3 GPS sats & 1 GLO sats	9.297	25.781	0.32%

**Table 2:** 3D position errors in short time for the first dataset (unit: meter).

Satellites Used	3 s	10 s	30 s	60 s
No sat	0.530	1.909	7.346	21.544
1 GPS sat	0.519	1.913	7.257	19.226
2 GPS sats	0.290	1.036	4.730	12.286
3 GPS sats	0.455	1.104	2.896	6.469
1 GPS sat & 1 GLO sat	0.380	1.684	6.830	17.676
2 GPS sats & 1 GLO sat	0.295	1.088	4.911	12.542
1 GPS sat & 2 GLO sats	0.592	1.999	5.828	10.622
1 GPS sat & 3 GLO sats	0.953	6.651	10.492	13.105
2 GPS sats & 2 GLO sats	0.577	2.146	5.944	10.744
3 GPS sats & 1 GLO sats	0.258	0.873	2.894	6.190

**Table 3:** Summary of horizontal errors using different satellites. GLO: GLONASS; sat: satellite.

Satellites Used	RMS (m)	Maximum Error (m)	Relative Horizontal Position Error
No sat	75.384	200.871	2.75%
1 GPS sat	101.945	263.778	3.73%
2 GPS sats	46.155	78.344	1.69%
3 GPS sats	3.248	4.945	0.12%
1 GPS sat & 1 GLO sat	79.460	208.918	2.91%
2 GPS sats & 1 GLO sat	35.668	56.542	1.31%
1 GPS sat & 2 GLO sats	28.046	55.919	1.03%
1 GPS sat & 3 GLO sats	5.509	13.952	0.20%
2 GPS sats & 2 GLO sats	4.935	11.791	0.18%
3 GPS sats & 1 GLO sats	2.878	4.956	0.11%

**Table 4:** 3D position errors at short times for the second dataset (unit: meter).

Satellites Used	3 s	10 s	30 s	60 s
No sat	0.475	2.663	20.109	58.576
1 GPS sat	0.451	1.847	20.245	45.248
2 GPS sats	0.446	1.930	10.080	20.443
3 GPS sats	0.402	1.146	3.967	7.058
1 GPS sat & 1 GLO sat	0.449	1.835	16.971	24.238
2 GPS sats & 1 GLO sat	0.444	1.920	9.800	17.631
1 GPS sat & 2 GLO sats	0.376	1.713	5.816	17.142
1 GPS sat & 3 GLO sats	0.330	1.078	3.526	5.763
2 GPS sats & 2 GLO sats	0.372	1.351	4.288	7.496
3 GPS sats & 1 GLO sats	0.399	1.134	3.875	6.684
